# Stigmas and Petals of *Crocus sativus* L. (Taliouine, Morocco): Comparative Evaluation of Their Phenolic Compounds, Antioxidant, and Antibacterial Activities

**DOI:** 10.1155/2024/6676404

**Published:** 2024-05-21

**Authors:** Asmaa Benkerroum, Khadija Oubella, Soukaina Zini, Kaoutar Boussif, Hind Mouhanni, Fouad Achemchem

**Affiliations:** ^1^Research Team Materials, Mechanical and Civil Engineering, National School of Applied Sciences, University Ibn Zohr, Agadir, Morocco; ^2^Bioprocess and Environment Team, LASIME Lab, Agadir Superior School of Technology, University Ibn Zohr, Agadir, Morocco

## Abstract

The dried stigmas of *Crocus sativus* L. produce saffron, a precious spice used for its culinary and medicinal properties since ancient times, while its petals are considered the main by-product of saffron production. The present study aimed to comparatively evaluate the phenolic content, antioxidant capacity, and antibacterial activity of methanolic extracts of stigmas and petals of *Crocus sativus* L. from Taliouine. The polyphenol content was measured using the Folin–Ciocalteu method, the antioxidant activity was determined using the DPPH free radical scavenging method, and the well-diffusion method was used to assess antibacterial activity against seven pathogenic bacterial strains (*Bacillus subtilis, Escherichia coli, Listeria monocytogenes, Proteus vulgaris*, *Pseudomonas aeruginosa, Salmonella enterica*, and *Staphylococcus aureus*). Furthermore, the minimum inhibitory concentration (MIC) of the extracts was determined using the microdilution broth test. Our findings revealed that stigmas and petals contained phenolic compounds at the rate of 56.11 ± 4.70 and 64.73 ± 3.42 mg GAE/g, as well as DPPH radical scavenging capacity with IC_50_ of 1700 *µ*g/ml and 430 *µ*g/ml, respectively. Petal extract showed more effective antibacterial activity, with inhibition diameters ranging from 10.66 ± 0.57 to 22.00 ± 1.00 mm and MIC values ranging from 2.81 to 5.62 mg/ml, compared to the stigma extract, which displayed inhibition diameters from 10.00 ± 0.00 to 18.67 ± 0.76 mm and MIC from 2.81 to 11.25 mg/ml, against five of the seven bacterial strains tested, including S. aureus, E. coli, P. vulgaris, P. aeruginosa, and *S. enterica*. Statistical analyses were performed to determine the significance of these results. Thus, stigmas and petals of *Crocus sativus* L. might serve as a suitable source of natural antioxidant and antimicrobial agents for application in the food and pharmaceutical industries.

## 1. Introduction

Aromatic and medicinal plants have always had a close relationship with humankind throughout the development of all ancient civilizations, serving various purposes in fields, such as cooking, perfumery, cosmetics, and medicine. Despite progress in the pharmaceutical field, the therapeutic use of plants remains a common practice in all countries, including Morocco [1]. According to the World Health Organization, almost 80% of the world's population in developing countries relies on plant extracts or their active ingredients to meet their primary healthcare needs [2]. Several considerable socioeconomic and ecological benefits are described in the scientific literature concerning the use of aromatic and medicinal plants, making their development a key objective in national agricultural development strategies.

In this context, research into the properties of aromatic and medicinal plants is more important than ever. Several studies have focused on medicinal plants as a valuable resource to identify new bioactive compounds with antioxidant properties, aiming to prevent complex diseases induced by oxidative stress, a primary contributor to most deaths, including cancers [3]. Additionally, research has also focused on the antimicrobial properties of these plants, in an effort to discover new natural antimicrobial agents with fewer side effects [4–6] and combat the emergence of antibiotic resistance in bacteria, due to the expanding use of chemical drugs [7]. Society itself is showing increasing interest in the benefits of alternative and complementary medicine based on aromatic and medicinal plants, as they are regarded as a more natural and less invasive means of treating and preventing disease than conventional medicine. Foremost among these plants is the *Crocus sativus* L. flower, which has been used for thousands of years to treat a variety of ailments and diseases [8]. This herbaceous autumn-blooming flower belongs to the Iridaceae family and the large *Crocus* genus, which includes over eighty species. *Crocus sativus* L. the only *Crocus* species that generates saffron by drying its red stigmas is *Crocus sativus* L.

The reproductive method of this particular species is through vegetative propagation using bulbs [12, 13]. It is cultivated in many places worldwide, such as Iran, India, Afghanistan, Morocco, and Euro-Mediterranean countries such as Greece, Spain, and Italy, with a world production of 418 t year 1 [14]. Iran has long been the world's largest producer and exporter of saffron, accounting for 85–90% of global production [15, 16].

According to the National Agency for the Development of Oasis and Argan Zones (ANDZOA), national saffron production is estimated at over 10 t in 2020, located in the Taliouine and Taznakht area, places Morocco fourth in terms of production after Iran, India, and Greece [12]. Saffron is the most expensive spice in the world, nicknamed “red gold.” Its labor-intensive cultivation takes around 150,000–200,000 (78 kg) fresh flowers and 160,000 petals to produce 1 kg of saffron [13], which explains its scarcity and high cost. In addition to their use in gastronomy, this spice has potential pharmaceutical and medicinal properties due to its richness in bioactive components [14], in particular, crocin, safranal, and picrocrocin, which contribute, respectively, to the color, aroma, and specific bitterness of saffron and have been identified as being responsible for these targeted therapeutic properties in the treatment of several diseases [15]. Antioxidant [16], anti-inflammatory, antitumor, antimicrobial, and antidiabetic priorities have also been described [17]. Clinical studies have indicated that saffron, in various doses, dose not present serious adverse side effects [17, 18]. Furthermore, in recent times, research has been undertaken to extract bioactive components not only from the stigmas but also from the petals of *Crocus sativus* L. Many studies have shown that these often neglected petals also contain interesting bioactive components [19, 20], mainly polyphenols, notably carotenoids, flavonoids, and anthocyanins, known for their antioxidant and antibacterial properties, as well as their multiple protective roles [21]. They have been associated with numerous pharmacological effects [22]. The results of previous studies have suggested that different parts of *Crocus sativus* L. can be used for their antioxidant activity [23, 24] and antibacterial effects against specific bacteria [12, 25, 26].

Our study aims to compare saffron, a highly esteemed spice, with its primary by-product, the petals of the endemic *Crocus sativus* L. variety from Taliouine, Morocco. We aim to evaluate their respective phenolic and antioxidant properties while also assessing their antibacterial activity against a range of resistant strains of pathogenic bacteria. To achieve this, we employed the methanol extraction method, known for its efficiency in maximizing the extraction of bioactive compounds. By undertaking this investigation, we seek to shed light on the potential of saffron and its petals as valuable sources of bioactive compounds with promising applications in various industries.

## 2. Materials and Methods

### 2.1. Plant Material

Stigmas and petals of *Crocus sativus* L. flowers, utilized in this study, were collected in October and November 2022 from the plant's natural biotope in Taliouine, Taroudant province, Souss Massa region, Morocco. Fresh samples were then shade-dried at room temperature and stored in a dark place awaiting analysis.

### 2.2. Preparation of Methanolic Extracts of Stigmas and Petals

Dried stigmas and petals were ground to powder and separately extracted by maceration with 80% methanol at an extraction ratio of 1 : 10 (v/w). To determine phenolic compounds and antioxidant activity, the macerate was shaken for 20 min at 350 rpm in the dark at room temperature and subsequently filtered through pleated filter paper (180-F, *Ø* = 150 mm, Dorsan, Barcelona). A second extraction was conducted under identical conditions, and the two filtrates obtained were combined and evaporated under vacuum using a rotary evaporator (IKA RV) at 45°C. The extract obtained was then reconstituted with 80% methanol and stored at 4°C [27].

To assess antibacterial activity, the macerate was shaken at 350 rpm for 24 h in the dark at ambient temperature. The resulting extract was then filtered and concentrated to dryness with the aid of a rotary evaporator. The concentrates obtained were then dissolved and taken up in 10% dimethyl sulfoxide (DMSO) at various concentrations of 45, 180, and 360 mg/ml and stored at 4°C until use.

The extraction yield of each extract derived from stigmas and petals of *Crocus sativus* L. was calculated according to the following equation:

(1) $$\begin{array}{c} Yield\;\%=\frac{W_{\text{dry extract}}\times 100}{W_{sample}}, \end{array}$$

where *W*_dry extract_ is the weight of each dry extract obtained in grams and *W*_sample_ is the weight of stigmas and petals' samples used in grams.

### 2.3. Bacterial Strains

Bacterial strains belonging to Gram-negative bacteria are *Escherichia coli* CECT 4076*, Pseudomonas aeruginosa* CECT 18*, Salmonella enterica* CECT 704, and *Proteus vulgaris* CECT 484 and to Gram-positive bacteria are *Staphylococcus aureus* CECT 976*, Listeria monocytogenes* CECT 4032, and *Bacillus subtilis* DSM 6633. These strains were provided by the Laboratory of Engineering Sciences and Energy Management, Agadir Higher School of Technology, Ibn Zohr University, Morocco.

### 2.4. Determination of Total Polyphenol Content

The total polyphenol content in methanolic extracts of stigmas and petals of *Crocus sativus* L. was determined using the colorimetric method, which relies on their capacity to reduce the Folin–Ciocalteu reagent, and gallic acid was used as a reference standard at various known concentrations, according to the protocol described by Wong et al., 2006, with a few modifications [28].

A volume of 0.5 ml of each prepared extract was combined with 1 ml of the Folin–Ciocalteu reagent (diluted 10-fold), and the mixture was thoroughly mixed and left to stand at room temperature for 5 min. Afterward, 0.8 ml of 7.5% Sodium Bicarbonate solution was added to the mixture, and it was left to incubate in the dark for 1 hour. Subsequently, the absorbance was measured at 760 nm using a UV-vis spectrophotometer (Agilent technologies). The results were expressed as milligrams of gallic acid equivalents (GAE) per gram of dry extract.

### 2.5. Determination of Antioxidant Activity

The antioxidant potential of methanolic extracts of *Crocus sativus* L. stigmas and petals was assessed through the 2,2-diphenyl-1-picrylhydrazyl (DPPH) assay. This widely recognized method evaluates the antifree radical potential of plant compounds or extracts by quantifying their capability to neutralize the chemical radical DPPH [29, 30]. DPPH is characterized by its dark violet hue. The strength of the extracts' antioxidant properties can be determined by how much color fading or decrease occurs when they are present.

For each extract, a volume of 0.5 ml was combined with 1.8 ml of a DPPH solution (0.1 mM). The mixture was thoroughly mixed and allowed in darkness at room temperature for 30 min.

Following the incubation period, the absorbance of each sample was measured at 517 nm against a blank utilizing a UV-vis spectrophotometer (Agilent Technologies) [31]. Triplicate measurements were conducted for each sample, and antioxidant activity is expressed as a percentage of DPPH inhibition, and wish is calculated using the formula as follows:

(2) $$\begin{array}{c} DPPH=\frac{\left({Abs}_{control}-{Abs}_{sample}\right)\times 100}{{Abs}_{control}}, \end{array}$$

where Abs_control_ is the absorbance of DPPH solution in methanol (Control). Abs_sample_ is the absorbance of the DPPH solution in the presence of the sample extracts tested.

Antioxidant activity is often expressed in terms of IC_50_ (half concentration inhibition), representing the extract concentration necessary to reduce the DPPH radicals by 50%. This value was determined through a graphical linear regression analysis of inhibition percentages against the various extract concentrations used [29].

### 2.6. Measurement of the Antibacterial Activity

Measurement of the antibacterial activity of *Crocus sativus* L. stigma and petal extracts at different concentrations was assessed using the agar well diffusion method, as outlined by Tagg and McGiven [32]. After checking the purity of the test organism, the bacteria were incubated at 37°C for a duration of 24 h in tryptone soy broth (TSB) (Biokar, Beauvais, France). The bacterial growth was adjusted to the 0.5 McFarland turbidity standard, corresponding to approximately 10^8^ CFU/ml. Subsequently, 15 ml of Mueller–Hinton agar (MHA) (Biokar, Beauvais, France) was aseptically poured into each Petri dish. Using a sterilized stainless-steel cylinder, 8 mm-diameter wells were made in the agar inoculated with the bacterial culture to be evaluated, and then each well was filled with 100 *μ*l of dilution of the prepared extracts. Chloramphenicol is employed as a positive control, while a 10% DMSO was included as a negative control. The Petri dishes were then placed in a refrigerator at 4°C for 1 h to facilitate the diffusion of the extracts into the medium. Following this, they were incubated at 37 ± 1°C for 16 to 24 h. The assessment of antibacterial activity involved measuring the diameter of the inhibition zone by use of a transparent scale and expressing it in mm. This test was repeated three times, and the average of the results was subsequently reported.

### 2.7. Minimum Inhibitory Concentration

Following confirmation of the antibacterial activity of methanolic petal and stigma extracts against tested bacterial strains, the MIC was determined using the microdilution broth sensitivity test, in line with the Clinical and Laboratory Standards Institute recommendations [33]. The extract was serially diluted in a 96-well microplate using the TSB medium. A standardized bacterial suspension adjusted to a concentration of 10^6^ cfu/ml was then added to each dilution; positive controls (culture medium with inoculum) and negative controls (culture medium without inoculum) were also included. After incubation at 37°C for 24 h, the well exhibiting the lowest extract concentration displaying no turbidity (indicating the absence of bacterial growth) was identified as the MIC, signifying that this specific extract concentration effectively inhibits bacterial growth. Each experiment was repeated three times.

### 2.8. Statistical Analysis

Statistical analysis was conducted using IBM SPSS (Statistical Package for the Social Sciences) version 25. Means and standard deviations were calculated, and statistical correlation between the various parameters tested for Crocus sativus L. extracts was assessed using Pearson's bivariate correlation test option. Results were deemed statistically significant if the *P* value was less than 0.01. In addition, principal component analysis (PCA) was carried out using ORIGIN Lab Pro 2017 software to further analyze and interpret the data.

## 3. Results and Discussion

### 3.1. Extraction Yield

The extracts of stigmas and petals of *Crocus sativus* L. from Taliouine used in our study were obtained using the 80% methanol maceration method. This extraction method gave yields ranging from 49.4 to 66% and from 45 to 64% for stigmas and petals, respectively. These yields vary according to the processing conditions and extraction methods used. Numerous studies have claimed that methanol extraction achieves higher extraction yields compared to other solvents, for *Crocus sativus* L [34], as well as for other plants [35].

### 3.2. Total Polyphenol Content

Phenolic compounds have been widely studied for their bioactive properties, particularly their protective effects against pathologies. A comparative analysis of total polyphenols in stigma and petal extracts of *Crocus sativus* L. from Taliouine was conducted using the Folin–Ciocalteu method. To facilitate this analysis, a standard calibration curve was generated (*y* = 0.0102 *x* + 0.0212 and *R*^2^ = 0.9951).

The results revealed higher levels of phenolic compounds in petals than in stigmas. The values in petals and stigmas were 64.73 ± 3.42 and 56.11 ± 4.75 mg GAE/g, respectively (Table 1).

The polyphenol content recorded in our study appears to be higher for Taliouine saffron stigma extracts than that described by Karimi et al. for Iranian saffron (6.45 mg GAE/g) [36]. However, it appears slightly close to that reported for the aqueous extract of Moroccan saffron stigmas (72.47 mg GAE/g) [37]. In our study, the phenolic compound levels found in the petal extracts of *Crocus sativus* L. were similar to those reported by Jadouali et al. and by Ouahhoud et al. for Taliouine saffron [38, 39]. They reported concentrations of 65.34 mg GAE/g DM and 64.66 mg GAEeq/g DM, respectively. However, the values obtained appear higher than those reported by Asgarpanah et al. for methanolic extracts of Iranian saffron petals (4.09 to 17.34 mg GAE/g MS) [40]. Although some studies have shown that petals of *Crocus sativus L.* contain more polyphenols than stigmas [39, 41], other studies have reported the opposite, with a concentration of 97.99 mg GAE/g for stigmas and 69.18 mg GAE/g for petals [42]. Thus, there is no clear consensus as to which of these floral parts of *Crocus sativus* L. contain the highest polyphenol content.

Indeed, extraction methods and other intrinsic and extrinsic factors could vary the phenolic compound content of plants. Moreover, the difference in polyphenol content recorded between organs of the same plant has been reported in many plants [43]. This variation reflects known variations in other secondary plant metabolites. It could also be interspecific and linked to the genotypic potential of plants in different countries, as has been described for wheat or according to the evolutionary stage or physiological state of the plants or according to the vegetative stage of the plants (vegetation or flowering period) [44]. Indeed, total polyphenol levels can vary quantitatively between leaves (fresh or dry), roots, stems, and flowers. In addition, intrinsic factors such as ecological and/or climatic conditions (temperature, sun exposure, drought, and soil) are known to condition the biosynthesis of secondary plant metabolites [45].

### 3.3. Antioxidant Activity by the DPPH Test

The antioxidant potential of methanolic extracts from the stigmas and petals of *Crocus sativus* L. was determined by the DPPH free radical scavenging test, which results in a characteristic color change from dark purple to pale yellow, indicating DPPH scavenging activity. This test showed a higher antioxidant activity potential for methanolic extracts of *Crocus sativus L*. than for aqueous and ethanolic extracts [36, 46]. The significant influence of solvent extraction power on polyphenol extraction yield and antioxidant power has been reported by numerous studies. Methanol appears to be particularly effective [47].

The DPPH scavenging activity of methanolic extracts of saffron stigmas and petals increased proportionally with extract concentration. A concentration of 1 mg/ml of extract gave a percentage inhibition of 34.74% for stigmas and 89.17% for petals (Table 1).

**Figure 1:**
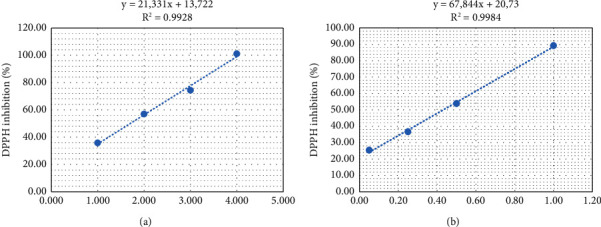
The percentage DPPH inhibition (%) in mg/ml of methanolic extracts: stigmas (a) and petals (b) of *Crocus sativus* L. from Taliouine, as a function of concentration.

The regression equations were used to calculate the IC_50_ indicating the free radical scavenging power of the extracts tested (Figure 1). The IC_50_ value obtained was 430 *µ*g/ml for the petal extract and 1700 *µ*g/ml for the stigma extract. A lower IC_50_ value indicates greater antioxidant activity; therefore, petals had a higher antioxidant capacity than stigmas and contained more antioxidant compounds.

These results are in line with those of the study by Ouahhoud et al. who reported that the radical scavenging capacity of petals was higher than that of stigmas, with an IC_50_ of 80.73 *µ*g/ml for petals and 1554.37 *µ*g/ml for stigmas [39]. In other studies, the IC_50_ for stigmas was estimated at 207.16 *µ*g/ml and 304 *µ*g/ml for ethanolic and aqueous extracts, respectively [48]. For petal ethanolic extracts, an IC_50_ of 504.26 *μ*g/ml was obtained by Jadouali Mohamed [49], while in the study by Wali et al., this value was 86.63 *µ*g/ml [50].

### 3.4. Antibacterial Activity

The antibacterial activity of methanolic extracts of stigmas and petals of *Crocus sativus* L. from Taliouine was assessed using the agar well diffusion method.

It is worth noting that various methods are available for determining bacterial susceptibility to antimicrobial agents, including disk diffusion, well diffusion, and broth or agar dilution. In our study, we opted the agar well diffusion method, as it is commonly recommended for highlighting the antibacterial effect of tested substances or extracts [51].

The results showed different degrees of inhibition by *Crocus sativus* L. stigma and petal extracts, depending on the pathogenic bacterial strains tested (Table 2).

**Figure 2:**
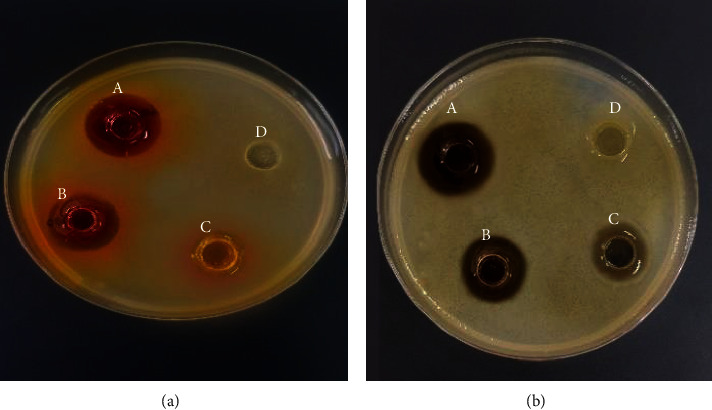
Antimicrobial activity of the methanolic extract from stigma (a) and petals (b) at various concentrations ((A) 360, (B) 180, and (C) 45 mg/ml) with (D) 10% DMSO, by the agar well diffusion assay.

Antibacterial activity against the strains tested increased significantly (*P* ≤ 0.01) with increasing concentrations of *Corcus sativus* L. extracts (Figure 2). Concentrations (45, 180, and 360 mg/ml) showed zones of inhibition ranging from 10.66 ± 0.57 to 22 ± 1.00 for petals and from 10 ± 0.00 to 18.67 ± 0.76 mm for stigmas against the following bacteria: *Staphylococcus aureus, Escherichia coli*, *Proteus vulgaris, Pseudomonas aeruginosa*, and *Salmonella spp*. The lowest concentration 45 mg/ml of the two extracts showed a slight inhibition of the growth of these seven bacteria, with zones of inhibition not exceeding 12.50 mm; however, this concentration in the stigma extract had no inhibitory effect on the growth of *Salmonella.*

The negative control (10% DMSO) showed no antimicrobial activity against any of the bacteria tested, while the positive control with chloramphenicol demonstrated efficacy against the tested bacteria.

According to these results, *Staphylococcus aureus* was the most sensitive, which concurs with the results obtained by Lachguer et al. [12]. However, no antibacterial activity was observed against *Listeria monocytogenes* and Bacillus subtilis, indicating resistance of these strains. The extracts demonstrated effective antibacterial activity against Gram-negative bacteria, E coli, P vulgaris, P aeruginosa, and *Salmonella*, than against Gram-positive bacteria, except for *S aureus.*

Results have been reported by Lahmass et al. that methanolic extracts of petals showed antibacterial activity against several strains, including *S. aureus* and *E. coli* [52]. In addition, several other studies have shown that saffron possesses antibacterial properties against several strains. Methanolic extracts were effective against Clostridium perfringens, Escherichia coli, Klebsiella pneumoniae, Pseudomonas aeruginos, Shigella flexneri, and Staphylococcus aureus [34], and petroleum ether and methanol extracts were effective against *Klebsiella pneumonia, Proteus vulgaris, Escherichia. coli, Staphylococcus aureus* and *Pseudomonas aeruginosa* [53]. Other studies have reported the antibacterial activity of aqueous extracts from Moroccan and Italian stigmas against bacterial strains *Escherichia coli, Salmonella typhimurium, Pseudomonas aeruginosa, Klebsiella pneumonia, Staphylococcus aureus*, and *Listeria monocytogenes* [37].

### 3.5. Minimum Inhibitory Concentration (MIC)

Determining the MIC is a crucial step in assessing the efficacy of antimicrobial agents, and dilution methods are considered the most appropriate for determining MIC values. These methods allow for the precise estimation of the concentration of the tested antimicrobial agent within the culture medium [51]. The microdilution technique involves preparing a series of diluted solutions of *Crocus sativus* L. stigma and petal extract in each well of the 96-well plate. The extract is tested at various concentrations, starting with a larger concentration in the first well and progressively lowering it in succeeding wells. After inoculating each well with a standardized bacterial suspension (10^6^ cfu/ml), the plates are incubated at 37°C for 24 h. The lowest concentration at which no bacterial growth is seen in a well is the MIC (Table 3).

Petals and stigmas extracts exhibited MIC values significantly higher (*p* < 0.01) than those of the positive control (chloramphenicol). The MIC values for petal extract were 2.81 mg/mL for *S aureus* CECT 976 and *P vulgaris* CECT 484 and 5.62 mg/mL for *E. coli* CECT 4076*, P. aeruginosa* CECT 18 and *S. enterica* CECT *704*. However, for stigma extracts, MIC values were 2.81 mg/mL for *S. aureus* CECT 976, 5.62 mg/mL for *E. coli* CECT 4076 and *P. vulgaris* CECT 484 and 11.25 for *P. aeruginosa* CECT 18 and *S. enterica* CECT *704*. In support of this, studies have reported that methanol and ethanol extracts of *Crocus sativus* L. demonstrated an MIC value of 6.5 mg/mL for *Staphylococcus aureus according* to Okmen et al. [54]. The MIC values in the study by *Asgarpanah* et al. were 31.2 mg/mL for Staphylococcus aureus, 62.5 mg/mL for *Salmonella*, and 125 mg/mL for *B. cereus* in *Crocus sativus* L. petal extract [40], while, according to Razavi and Jafari, the inhibitory effect of methanolic extracts of stigmas showed MIC values lower than the results of the present study, ranging from 0.33 to 1.23 mg/mL against *E. coli, P. aeruginosa*, and *S. aureus* [55].

Based on the results obtained, it was observed that extracts from the petal of *Crocus sativus* L. were more effective in inhibiting bacterial growth when compared to extracts from the stigma. Furthermore, these extracts were found to have a more marked effect on Gram-negative bacteria than on Gram-positive bacteria. This difference in sensitivity is due to differences in bacterial cell structure and composition.

Let uss recall that *Crocus* sativus L. possesses various phytochemical properties that have been linked to its antibacterial activity; this activity has been attributed to the presence of compounds such as safranal and crocin [55–57], as well as flavonoids such as kaempferol, quercetin, and an isorhamnetin derivative [58]. These compounds are volatile or water-soluble, enabling them to easily reach contaminating microorganisms and contribute to their destruction [59].

The variability in antimicrobial potential recorded by the authors is linked to several direct and indirect factors that accompany the sample from implantation to the final product. These factors include the age of the corms [55], altitude and climate [60], drying temperature [61], storage and packaging [62], and extraction and analysis methods [36, 63]. All these factors can affect the chemical composition of the sample and therefore the results obtained during analysis. Consequently, it is important to consider these factors and control them as much as possible to minimize the variability of results and obtain reliable and reproducible data.

### 3.6. Statistical Analysis

#### 3.6.1. Correlation between TPC, Antioxidant Capacity, Antibacterial Activity, and MIC

The results of the statistical measurements analyzed of *Crocus sativus* L. extracts indicated significant positive correlations according to Pearson's correlation analysis at a significance level *p* < 0.01 between the different parameters analyzed.

Stigma extracts showed a significant positive correlation between a zone of inhibition and MIC (*r* = 0.926). In addition, a strong positive correlation was observed between total polyphenol content and antioxidant activity, with a correlation coefficient (*r* = 0.962) (Table 4). These results were in line with those reported by Acar et al. [64]. For the petals, a positive correlation was observed between the zone of inhibition and MIC (*r* = 0.723). There was no significant correlation found between total phenolic content and antioxidant activity (Table 5). This finding is consistent with the results of previous studies for other medicinal plant extracts [65, 66], indicating that antioxidant capacity and total phenolic content were not correlated. This may be explained by the fact that, in addition to phenolic compounds, other phytochemicals contained in *Crocus sativus L.* extracts, such as flavonoids, carotenoids, and vitamins, may contribute synergistically to this overall antioxidant capacity. On the other hand, determination of the total phenolic compound content using the Folin–Ciocalteu method does not represent an absolute measure of the quantity of phenolic compounds.

#### 3.6.2. Principal Component Analysis (PCA)

**Figure 3:**
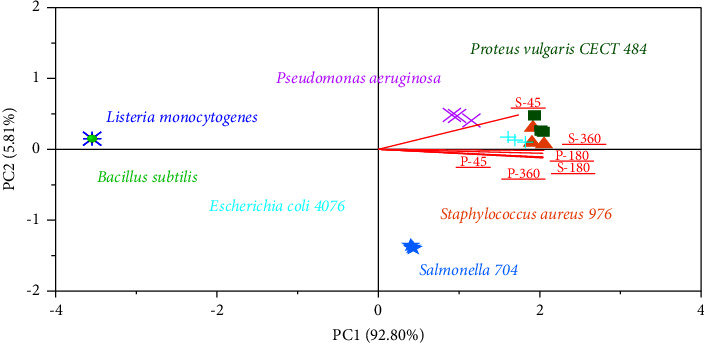
PCA effects of different concentrations of *Crocus sativus* L stigma and petal extracts against bacterial strains.

The effect of concentrations (45, 180, and 360 mg/ml) of petal and stigma extracts on the antibacterial activity of the different strains assessed was assessed using the statistical technique of principal component analysis (PCA) (Figure 3). Principal component 1 (PC1) contributed 92.80%, while principal component 2 (PC2) accounted for 5.81% of the total variability of the components represented. According to the results, similarities were highlighted by the small distance between the concentrations of stigma and petal extracts, suggesting that these extracts have significant effects in terms of zone of inhibition against bacteria (Staphylococcus aureus, Escherichia coli and Proteus vulgaris, Pseudomonas aeruginosa, and *salmonella*). In addition, the 360 mg/ml concentration was associated with the highest antimicrobial response, and the activity increased with the increasing concentration. *Staphylococcus aureus* proved the most sensitive one. However, a slight dissimilarity was observed with *Salmonella*, which proved less sensitive to extracts than the other bacterial strains evaluated.

## 4. Conclusion

The extracts obtained from the stigmas and petals of *Crocus sativus* L., originating from Taliouine in the Taroudant Province of Morocco, exhibit a significant abundance of bioactive compounds, particularly polyphenols. These floral components demonstrate remarkable antioxidant and antibacterial activities, thereby offering protective effects against cellular oxidation processes and pathogenic microorganisms. The presence of these valuable properties in *Crocus sativus* L. from Taliouine highlights the underappreciated potential of saffron and its petals. The outcomes of this study open new avenues for the extensive utilization of *Crocus sativus* L. in diverse fields, including the pharmaceutical, food, and cosmetics industries. The incorporation of *Crocus sativus* L. and its derived products holds promise for the development of innovative and effective solutions in these domains, capitalizing on their inherent bioactive properties.

## Figures and Tables

**Table 1:** Total polyphenol content and antioxidant activity of methanolic extracts of stigmas and petals of *C. sativus* L. Taliouine (*n* = 3).

Sample	TPC (*µ*g/ml)	TPC (mg GAE/g)	DPPH inhibition (%) (at a 1 mg/mL concentration)	IC_50_ (*µ*g/ml)
Stigmas	168.34 ± 14.25	56.11 ± 4.75	34.73 ± 0.28	1700 ± 23.06
Petals	323.69 ± 17.11	64.73 ± 3.42	89.17 ± 1.92	430 ± 19.46

TPC: total polyphenol content; GAE: gallic acid equivalents; IC_50_: 50% inhibitory concentration.

**Table 2:** Antibacterial activities of methanolic extracts of stigmas and petals of *Crocus sativus* L from Taliouine.

	Flower organ	Inhibition zone (mm)						
		Petals			Stigmas			Negative control
	Extract concentration (mg/ml)	360	180	45	360	180	45	
Bacterial strain	*Staphylococcus aureus* CECT 976	**22.00** **±** **1.00**	20.66 ± 1.04	12.00 ± 1.00	**18.67** **±** **0.76**	13.00 ± 1.00	10.33 ± 0.57	—
	*Escherichia coli* CECT 4076	20.33 ± 0.57	18.33 ± 0.57	12.50 ± 0.50	17.00 ± 0.57	13.66 ± 0.28	10.00 ± 0.00	—
	*Proteus vulgaris* CECT 484	21.66 ± 0.57	19.66 ± 0.57	11.33 ± 0.28	18.67 ± 0.58	14.33 ± 0.57	11.33 ± 0.75	—
	*Pseudomonas aeruginosa* CECT 18	15.33 ± 1.15	12.66 ± 1,15	10.66 ± 0.57	17.66 ± 0.57	12.00 ± 0.00	10.00 ± 0.00	—
	*Salmonella enterica* CECT *704*	18.33 ± 0.57	14.50 ± 0.50	10.66 ± 0.28	14.33 ± 0.57	12.33 ± 0.57	—	—
	*Listeria monocytogenes* CECT 4032	—	—	—	—	—	—	—
	*Bacillus subtilis* DSM 6633	—	—	—	—	—	—	—

Values in bold indicate the highest inhibition zones, these values can be left in their usual format.

**Table 3:** MIC of methanolic extracts of stigmas and petals of *Crocus sativus* L. from Taliouine.

Bacterial strains	MIC mg/ml		
	Petals	Stigmas	Chloramphenicol
*Staphylococcus aureus* CECT 976	2.81	2.81	0.015
*Escherichia coli* CECT 4076	5.62	5.62	0.008
*Proteus vulgaris* CECT 484	2.81	5.62	0.011
*Pseudomonas aeruginosa* CECT 18	5.62	11.25	0.011
*Salmonella enterica* CECT *704*	5.62	11.25	0.008
*Listeria monocytogenes* CECT 4032	—	—	0.015
*Bacillus subtilis* DSM 6633	—	—	0.015

**Table 4:** Linearity among different analyzed parameters of *Crocus sativus L.* stigmas.

	Polyphenols	IC_50_	Inhibition zone	MIC
Polyphenols	1			
IC_50_	**0.962** ^*∗∗*^	1		
Inhibition zone	0.017	0.015	1	
MIC	0.000	0.000	**0.926** ^*∗∗*^	1

^*∗∗*^Correlation is significant at the 0.01 level (two-tailed). Values in bold in the table indicate significant correlations.

**Table 5:** Linearity among different analyzed parameters of *Crocus sativus L.* petals.

	Polyphenols	IC_50_	Inhibition zone	MIC
Polyphenols	1			
IC_50_	0.396	1		
Inhibition zone	0.004	0.013	1	
MIC	0.000	0.000	**0.723** ^*∗∗*^	1

^*∗∗*^Correlation is significant at the 0.01 level (two-tailed). Values in bold in the table to indicate significant correlations.

## Data Availability

The data used to support the findings of this study are available from the corresponding author upon request.
